# Experiences, perspectives and expectations of adolescents with juvenile idiopathic arthritis regarding future work participation; a qualitative study

**DOI:** 10.1186/s12969-020-00429-6

**Published:** 2020-04-15

**Authors:** E. Charlotte van Gulik, Floris Verkuil, Anouk M. Barendregt, Dieneke Schonenberg-Meinema, Amara Nassar-Sheikh Rashid, Taco W. Kuijpers, J. Merlijn van den Berg, Jan L. Hoving

**Affiliations:** 1 Emma Children’s Hospital, Amsterdam UMC, University of Amsterdam, Pediatric Immunology Rheumatology and Infectious diseases, Location AMC | G1-215 | Meibergdreef 9, Amsterdam, 1105AZ The Netherlands; 2 Amsterdam UMC, Department Coronel Institute of Occupational Health. Amsterdam Public Health research institute, P.O. Box 22700, Amsterdam, DE NL-1100 The Netherlands; 3 Research Center for Insurance Medicine (KCVG), Amsterdam, The Netherlands

**Keywords:** Juvenile idiopathic arthritis, Adolescents, Vocational support, Qualitative research

## Abstract

**Background:**

Having Juvenile idiopathic Arthritis (JIA) has widespread implications for a person’s life. Patients have to deal with recurring arthritis, characterized by pain often accompanied by a loss of energy. Since JIA often persists into adulthood, patients with JIA are likely to encounter difficulties in their working life. We expect that the experiences in school life may be comparable to the barriers and opportunities which patients affected by JIA encounter in adult working life. Therefore, the aim of this study was to elicit the experiences during school life and the perspectives and expectations regarding future work participation of adolescents with JIA.

**Methods:**

This study used individual, semi-structured interviews and followed a predefined interview guide. Participants between 14 and 18 years of age (*n* = 22) were purposively selected to achieve a broad range of participant characteristics. Open coding was performed, followed by axial coding and selective coding.

**Results:**

Great differences were seen in the support and understanding that adolescents received in dealing with JIA at school, leisure activities and work. Varying approaches were mentioned on how to pursue a desired vocation. Perspectives regarding disclosure varied. Participants wished to be approached like any other healthy adolescent. Expectations regarding work participation were positively expressed.

**Conclusion:**

This study showed that participants often disregarded having JIA when making plans for their future career. Facilitating an open discussion about the possible limitations accompanying JIA with educators and employers might prevent overburden and increase the chance of starting a career which would accommodate the patient with JIA in the near and distant future.

## Background

Having juvenile idiopathic arthritis (JIA) has widespread implications in a person’s life. JIA encompasses all forms of arthritis that begin before the age of 16, persist for more than 6 weeks, and are of unknown etiology [1]. Recurring arthritis is the hallmark of this disease, characterized by pain and loss of function and is frequently accompanied by loss of energy [2]. Since JIA often persists into adulthood [3–5], patients with JIA are likely to encounter difficulties in their working life. Earlier studies showed inconsistencies regarding employment rates and educational achievements of patients with JIA in comparison to the general population [6–11]. This ranges from no differences in occupational outcomes between JIA and comparison groups [7] to an alarming 32% of patients with JIA who are either disabled, receive a disability pension, are on sick leave or undergoing rehabilitation treatment [10]. Consequences of chronic illness in work and employment pose a great burden on both the individual and society. Therefore, prevention of work disability has increasingly become a topic of scientific research [12–15].

In vocational psychology the terms “aspirations” and “expectations” are used to denote the difference between what someone wants to achieve or become and what someone thinks is possible to achieve given their limitations and capabilities. Vocational development is presented as a life-long process, beginning in early childhood [16, 17] leading to ‘vocational adaptability’ [18], which is defined as: “the readiness to cope with the predictable tasks of preparing for and participating in the work role and with the unpredictable adjustments prompted by changes in work and working conditions”.

We question whether experiences of patients with JIA during school life influence aspirations and expectations of their career.

To reach vocational adaptability, vocational support is important but rated far from satisfactory by adolescents with JIA [19]. Low expectations from others and limited career advice are seen as important barriers during career development [19]. We hypothesized that these barriers in the early career of JIA patients could be avoided, if proper support would be available. From whom and to what extent patients with JIA need support regarding their future career is as of yet unknown.

We expect that the experiences of patients affected by JIA during their school life, including leisure activities and part-time jobs, may be comparable to the barriers and opportunities which they encounter in adult working life. Therefore, the aim of this study was to elicit the experiences during school life and the perspectives and expectations regarding future work participation of adolescents with JIA.

## Patients and methods

### Design

The Consolidated criteria for reporting qualitative research (COREQ) checklist [20] was used for comprehensive reporting of this study. Using individual face-to-face interviews of 1 hr we interviewed patients with JIA at the Amsterdam UMC, location Academic Medical Centre (AMC) between April 2017 and January 2018. This qualitative study was performed in accordance with the Declaration of Helsinki and approved by the institutional review board (reference number W17_004#17.016). Informed consent of the patient was recorded and signed by the patient. When the patient was under the age of sixteen, both parents and patient signed the informed consent. The interviews were semi-structured [21] and followed a predefined interview guide (Table 1). Probe questions were used to gather more specific details on the experiences, perspectives and expectations of JIA patients. The interview guide was part of the patient information form and mailed to participants in advance to prepare for the interview.

**Table 1:** Interview guide for individual interviews

Opening question:	
What are your plans after graduation?	
Main questions:	
What are your perspectives and expectations regarding your future work participation?	
How do you think the disease will influence your career?	
Regarding:	
Choosing career / further education	
Finding a job / further education	
Applying for a job / further education	
Holding a the job / finishing your education	
Additional questions:	
How does/did the disease influence your time at school?	
Are/were there adjustments made to support you?	
Do you have a part time job?	
What is your experience with working with your illness?	
Closing question:	
Where do you see yourself ten years from now?	

The interviews took place without parental attendance and were scheduled just before or after a planned visit to the hospital to minimize inconvenience. Participants were asked if they agreed on being contacted by telephone if additional questions would arise.. When appropriate field notes were made after the interview. The interviewer was a female medical doctor and PhD candidate [ECvG] trained in conducting qualitative research and supported during the first two interviews by a senior researcher [JLH] experienced in conducting qualitative research. The interviewers were not involved in the medical care of the participants. To minimize a potential bias regarding data interpretation, experiences and personal beliefs of the interviewer were reflected upon within the research group prior to and during data collection.

### Patient selection

Participants were purposively selected to achieve a broad range of participant characteristics, including age, gender, educational level, time since diagnosis of JIA and symptom severity. Therefore a list was derived from the outpatient clinic agenda and eligible patients were contacted by mail and subsequently by phone by one of the researchers. Reasons for refusal were noted. In order to be eligible to participate in this study, a subject had to meet the following criteria: 1. Diagnosed with JIA according to the ILAR (International League Against Rheumatism) categories at least 6 months prior to the start of the study; 2. Between 14 and 18 years of age; 3. Dutch-speaking; 4. Informed consent by patient and, when the patient was under the age of 16, from the caregiver/parent as well. Participant characteristics, as defined below, were evaluated to determine if our patient cohort was sufficiently diverse. Patients were invited on a rolling basis until data saturation was reached.

### Data collection

Gender, age, age at diagnosis, JIA subtype (according to the ILAR classification) and disease activity status, as recorded by the treating pediatric rheumatologist, were collected from the patient record. Disease activity status was divided in clinical remission (on and off medication combined), clinical inactivity as defined by Wallace [22] and clinically active disease (not fulfilling the Wallace criteria). Additionally, the current level of education was noted. Before the interview started, the participant was asked to fill out 1) the Dutch version of the Child Health Assessment Questionnaire (CHAQ) as a measurement of functional ability in daily activities on a scale from 0.000 to 3.000, where lower scores indicate better functional ability [23] and 2) the Pediatrics Quality of Life inventory (PedsQL), which measures health related quality of life on a scale from 0 to 100, where higher scores indicate a higher health related quality of life [24]. Interviews were held until data saturation was reached, with a minimum of 20 individual interviews. The acquired data were safely stored using encrypted files which were not available outside the hospital network.

### Data analysis

All interviews were audio recorded and transcribed *verbatim*. Open coding was performed, followed by axial coding and selective coding as presented by Strauss and Corbin as grounded theory approach [25]. Themes were derived from the data organised in the three topics: experiences, perspectives and expectations. Analysis started after the first interview and continued throughout the study. Three interviews were coded independently by two researchers in a blind fashion and discussed afterwards. These discussions allowed for different views and interpretations in order to reach consensus regarding the code system used. If necessary, phrases were coded multiple times during the open coding phase. For coding MAXQDA Plus 12 (VERBI GmbH Berlin/Germany) was used. For descriptive statistics, SPSS software version 24 (IBM Corp., Armonk, NY) was used.

## Results

Thirty-one eligible patients were contacted to participate in this study. One patient refused because of unpleasant experiences with participating in research in the past, six patients did not feel like participating and two patients could not fit it into their schedule. No repeat interviews were conducted. A total of 22 patients were included in this study, 6 males and 16 females with a median age of 16.7 years (range 15–17), a median CHAQ of 1.000 (range 0.000–2.250 on a scale from 0.000 to 3.000) and a median PedsQL Life inventory of 78.5 (range 35–94). The ranges of both CHAQ and PedsQL indicate a broad range of experienced limitations and complaints of JIA in daily life. Eight participants had inactive disease, two participants had JIA in remission and 12 participants had active disease. The individual patient characteristics are shown in Table 2. Participants were enrolled in different levels of secondary education, including prevocational and higher secondary education, pre-university education and secondary vocational education. All participants but one had or had had a part-time job during evening hours or in the weekend. The physical demands of these jobs ranged from shifts of 2 hrs as a cashier to 6 hrs doing the dishes at a restaurant.

**Table 2:** General characteristics per participant

Participant number	Age (years)	Gender	Diagnosis	Age at disease onset (years)	Activity status	CHAQ	PedSQL
1	15	Male	ERA	13	active	0,625	73
2	16	Male	Oligo articular	5	inactive	0,125	93
3	17	Female	Poly articular RF -	12	active	1880	57
4	16	Female	Poly articular RF -	14	active	2250	54
5	17	Male	ERA	8	active	1125	80
6	16	Male	Systemic JIA	14	active	0,000	86
7	17	Female	Poly articular RF -	1	remission	0,000	86
8	16	Female	ERA	11	inactive	0,875	78
9	17	Female	Poly articular RF +	13	inactive	1250	57
10	17	Female	Poly articular RF -	9	inactive	2250	68
11	17	Female	Oligo articular	14	inactive	1125	87
12	17	Female	Oligo articular	8	active	1000	79
13	15	Female	Oligo articular	12	remission	0,125	93
14	17	Female	Poly articular RF +	14	active	0,125	94
15	17	Male	Psoriatic arthritis	4	active	0,500	89
16	17	Male	Poly articular RF -	14	active	1500	88
17	16	Female	Poly articular RF -	2	inactive	0,125	83
18	16	Female	Poly articular RF -	5	inactive	1000	61
19	15	Female	Oligo articular	1	inactive	0,250	70
20	16	Female	Poly articular RF +	13	active	2250	35
21	16	Female	Poly articular RF -	6	active	1500	57
22	16	Female	Poly articular RF -	13	active	1500	41

-: negative; +: positive; *CHAQ* Childhood Health Assessment Questionnaire, *ERA* enthesitis related arthritis, *PedsQL* Pediatric Quality of Life Inventory, *RF* rheumatoid factor

**Fig. 1:**
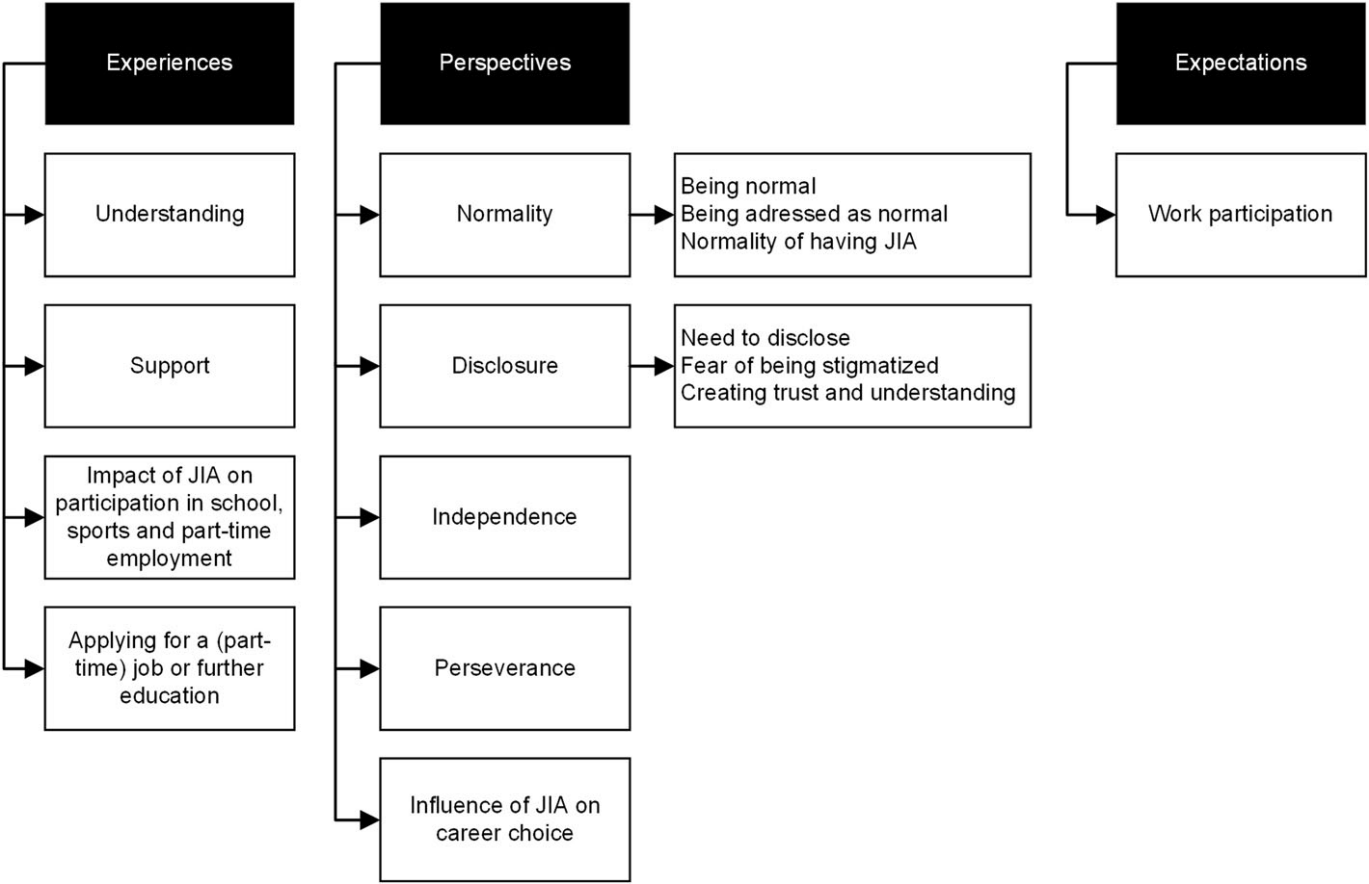
Coding tree

Main themes and subthemes were arranged according to patients’ experiences (having JIA in school life and part-time jobs), perspectives (regarding future work participation) and expectations (regarding future work participation). The coding tree is depicted in Fig. 1.

### **Experiences with having JIA in school life and a part-time job**

Associated interview excerpts can be found in Table 3.

**Table 3:** Experiences, subthemes and associated interview excerpts

Quotation	Illustrative quotes	Participant nr
***Subtheme: Understanding***		
1	There will always be a teacher who responds to me being tired, with the words: “I also get tired, but still got to do stuff.” And then I think, it is just not the same.	8
2	Sometimes I couldn’t take a test because I was sick or had to go to the hospital and everyone was like “she can always skip tests and this and that”. They knew I have JIA, but in time they seem to forget, because you can’t see it. So they don’t understand.	20
3	My teacher was really... he has a sister who has a rheumatic disease, so he really understood what was going on.	3
***Subtheme: Support***		
4	Eventually, I got an extra set of schoolbooks, but I had to pay for everything myself and had to organize it myself. They told me they had no funds. Only after multiple contacts between school and the rehabilitation centre, suddenly things were possible.	9
5	They set up a room for me with a couch, when I was tired I could go and lie down for an hour. I didn’t partake in gym class, I only had to go to classes of which the subject came up in my exams. I had permission to work on a laptop, therefore all my books were on the laptop. They offered school transport, but then I had to get up 45 min earlier, so I rejected.	15
6	My parents were always there and always accompanying me to the hospital. They support me in everything, school, studying. They make sure everything’s staying on the right track.	8
7	My friends especially, they are really worried, like: “you can’t do this, you shouldn’t do that”, which is sweet, but I’m more like, I can decide for myself.	18
***Subtheme: Impact of JIA on participation in school, sports and part-time employment***		
8	I’ve always had less energy, especially last year. Sometimes I’m even unable to do my homework, because I’m too tired after school and all I want to do is sleep. Or when I try to do my homework, I just fall asleep studying.	8
9	I started at the highest level, but last year I had to step down, because the high level and the JIA and fatigue just didn’t work.	22
10	I played hockey a lot. I played in the preselection team, but then I got JIA and had to stop. During two to 3 years, I could not participate in any sport.	9
11	“The friend with whom I worked, knew I had it, so he was very considerate. He lifted the most heavy parts.” (Working in a garden centre)	5
12	They arranged a switch with a colleague so I could help with the lessons and feeding in the evening instead of mucking out stables in the morning. My colleagues are my friends so they knew I have JIA and they didn’t mind. (Working in an equestrian facility)	4
13	At this moment I’m working with my sister at the cinema and I have told about my JIA. So when I’m not able to do something, they’ll take over.	9
***Subtheme: Applying for a (part-time) job or further education***		
14	They thought I wasn’t up for the job, that I wouldn’t be able to make long hours or stand for a longer period. If that was the case, they couldn’t use me and I just didn’t got the job.	11

### Understanding

Participants encountered indifference or disbelieve regarding the impact JIA can have in school attendance and performance. This was attributed to the variable course and often invisible character of the disease. Patients experienced a lack of understanding from fellow students as well as teachers (quotations 1 and 2).

On the other hand, when people had a rheumatic disease themselves or knew someone with a rheumatic disease, consideration and empathy was felt to be evident and genuine.

### Support

Participants mentioned different forms of support. Most participants received practical support, e.g. ergonomic adjustments at home, school and at work. Practical support at school was arranged in different ways (quotations 4 and 5). Most schools offered an elevator pass and a second set of school books to lighten the physical burden or provided extra time during examinations when writing was difficult. However, there were differences in the way schools handled applications for (financial) support. Some participants reported great difficulty in gaining the needed support (quotation 4).

Medical support was described by participants as providing medical care, rehabilitation or granting a medical certificate. Some participants thought that the impact of JIA on school or a part-time job as well as the other way around, should be discussed with the paediatric rheumatologist. Participants wanted to discuss what they should or should not do to improve the course of the disease or to prevent a flare. Some participants wanted their paediatric rheumatologist to write a medical certificate to help them get support and understanding at school or work. Psychological support, i.e. providing the possibility to talk about what it means to have JIA and how to deal with JIA, was regarded as either helpful or unnecessary.

Two other forms of support were related to the attitude of others: being supportive, i.e. providing encouragement and understanding and ‘being there’; or being protective, i.e. being vigilant, warning for possible impact of certain activities on disease activity. The appreciation of these differed. A supportive environment (e.g. family and friends) was deemed essential, but a protective attitude, specifically from family members was experienced as overbearing at times (see quotations 6 and 7).

### Impact of JIA on participation in school, sports and part-time employment

All participants were occasionally absent from school due to JIA. This differed from weeks on end to once every 3 months due to hospital appointments. Lack of energy and pain were frequently reported reasons to stay at home and limited the ability to do homework (quotation 8). JIA activity forced a few participants to cut back workload for school activities and also resulted in grade repetition or switching to a lower educational level (quotation 9). Some participants were not able to maintain a high level of sports due to JIA activity (quotation 10).

Some stated that complaints were not always taken seriously during a part-time job, while for others adjustments were made in the workload. Workload adjustments were easily implemented when colleagues were friends or family, as these were considered more supportive (quotations 11–13).

### Applying for a (part-time) job or further education

Both negative as well as positive experiences were mentioned when participants applied for a part-time job or further education. Negative experiences included not being hired for a part-time job (quotation 14), not being accepted for further education and having to convince an admissions officer that he or she was able to complete the chosen educational programma. Positive experiences included gaining reassurance from the employer that it would work out and when an employer stated that he/she did not mind the fact that the employee had JIA.

### **Perspectives**

Associated interview excerpts can be found in Table 4.

**Table 4:** Perspectives, subtheme ‘Normality’ and ‘Disclosure’ and associated interview excerpts

Quotation	Illustrative quotes	Participant nr
***Subtheme: Normality***		
15	When I had to bring many books with me, it would cause pain in my shoulders. I could have applied for a second set of books, but then I would be so different from the others which I’m rather not.	11
16	The school doctor asked me if I needed any adjustments. I told I had a pillow and a laptop and such on high school but that I don’t want any of it anymore. I just want to live a normal life.	5
17	I want them to see me as I am, I don’t want them to treat me as someone who isn’t capable of anything.	11
18	People often think I’m weak, that I will give up easily because I’m not able to do everything.	3
19	The reason I’m not telling people, is because they immediately think I’m pathetic you know, I don’t like that at all. I don’t want to be pitied.	22
20	I think it’s unnecessary to make such a big deal out of it. I’ve got JIA and I don’t think it’s a big deal so you shouldn’t think so either.	19
***Sub-theme: Disclosure***		
21	It depends on how I’m feeling. If I have a lot of complaints, of course I’ll tell, otherwise they can’t take it into account. But like now, I don’t think it’s necessary.	5
22	When I’m graduated and I’m really going to work fulltime, I’ll tell straight away. Because you’ll work there every day and I might have to visit the hospital or something like that, then it’s important that they’ll know. But with a part-time job …	16
23	“I think I’m less likely to be employed than someone who doesn’t have it. Because, they might think I won’t be able to walk for a long period or something like that, though I can do that easily.”	6
24	I know for sure that they’ll think: “this is not someone we want to keep” … Because they rather not employ someone who’s sick, I’m certain of it.	5
25	I think it will only work in your disadvantage, when you don’t tell having complaints. I think they (red. The employer) would appreciate you more if you do tell. And yes, I think they should know.	8
26	If you tell straight away, then they’ll know from the start. Then if they hire you, they trust it will work out and then it’s ok. I think a company will have more faith in you from that point.	2
***Subtheme: Independence***		
27	It is my body, so I think, I should decide	12
28	I might think about it, but if I think I can manage, I will listen to myself. I know my body better than anyone else.	18
29	I will not become dependent of anyone else	9
30	I think that the only person I need, is me.	13
31	I knew I would be exhausted during the whole weekend. For example, when I had a party on Friday, I knew I couldn’t plan anything on the other days because I would need to rest.	17
32	I asked the paediatric rheumatologist for a medical certificate and brought it with me to school and then I asked if it would be possible to shorten my school days because of my JIA.	10
33	I advise to just organize things yourself and not just wait for something to happen. I’ve learned to ask for the things I need and to say when I need help instead of just ploughing on.	18
***Subtheme: Perseverance***		
34	I know I’m going to be in pain, but I’ll accept it, since I’m not going to put everything aside.	11
35	Most of the time I notice directly (red. I pushed the limit) and then I’ll probably continue for some time. The day after, it’ll be much worse. And then I think: this wasn’t a smart move. But then again, I had fun and I’ll stay happy. Because when you’re always in pain, staying at home does not do you any good. You’ll become completely secluded.”	21
36	I’m always attending school, even in my wheelchair, even on crutches	11
37	At this moment, I’m trying to attend school every day, because it’s really difficult when I’m missing out on class. Even when I’m absent for only one day, I’ll miss out on so much. But I’m always trying to go to school, but sometimes it happens, I just can’t.	8
38	I will do my job, even if I have to push through the pain, I don’t mind.	1
***Subtheme: Influence of JIA on career choice***		
39	I feel really passionate about this. And if you really want something, your body can try to stop you, but if you know for sure, you’ll just keep going	3
40	If it’s going to cause pain, I can always quit. But if I don’t try it, I won’t know for sure.	17
41	To me it doesn’t matter whether I have JIA or not, if I want to do something, I will.	12
42	I’ll look for something that I would like and want to do. Of course I’ll pay some attention to what’s possible, but if I’ll take that seriously, I’m not able to do anything.	20

### Normality

Normality is a theme which arose in three different connotations. The wish for being normal, being addressed as a normal person and the normality of having JIA. The wish for being normal was expressed in wanting to be capable of working without any adjustments and not showing any weakness in comparison to others. It was brought up as a reason to refuse ergonomic adjustments, see quotations 15 and 16. Most of the participants expressed the desire for being addressed as a normal adolescent and not as a patient. That was often the reason for not disclosing having JIA to new classmates or colleagues (quotations 17–19). Despite the variable course of the disease, having JIA had become the normfor most of the participants, especially for those who had been diagnosed with JIA in early childhood or multiple years ago (quotation 20).

### Conflict of interest

Participants reflected on whether they would tell their future employers about JIA. Different reasons for disclosing or concealing having JIA are given and can be categorized in three subthemes: need to disclose, fear of being stigmatized and creating trust and understanding.

#### Need to disclose

If participants felt the need to disclose having JIA to a future employer the severity of complaints and the work position were taken into consideration. Quotation 21 serves as an example of how complaints were taken into account in the need to disclose. When complaints were severe, participants indicated that disclosure was necessary to gain respect and consideration from the employer. When participants expected that JIA would not influence work participation in the near future, they were disinclined to disclose having JIA. Another differentiation was made between applying for a temporary or a permanent position. Participants mentioned that they felt less obliged to disclose having JIA when applying for a temporary position than when applying for a permanent position (quotation 22). Because it was a delicate subject to address, some participates mentioned that if the employer would ask, they would answer truthfully, but would not spontaneously disclose having JIA themselves.

#### Fear of being stigmatized

Some participants claimed they would not disclose having JIA in the future. They did not want to be treated as a patient, or as someone who should be pitied. Consequently, they feared being rejected because of JIA (quotations 23 and 24).

#### Creating trust and understanding

Disclosure of having JIA was also described as being honest and therefore starting a career based on mutual trust between employee and employer (quotations 25 and 26).

### Independence

Independence is described as making your own decisions (i.e. becoming autonomous), planning your activities while considering JIA and organizing the required support yourself. Participants considered themselves as the person who should always be in charge, as quotations 27–30 illustrate. Participants often planned their activities, weighing the advantages and disadvantages of participation in regard to their complaints which would consequently arise, see quotation 31. Quotation 32 is an example of how a participant arranged the required support. Quotation 33 underlines the importance of being proactive in arranging the required support.

### Perseverance

Participants reported that they constantly had to consider pain and tiredness as a consequence of participating in activities. In some situations participants found it necessary to plan activities while taking their complaints in account. Additionally, the mind-set “just keep going, regardless of the pain” was mentioned as a way to cope with JIA and to maintain a social life (quotations 34–38).

### Influence of JIA on career choice

The responses from participants on the influence of JIA on career choice varied from abandoning their initial career choice owing to JIA, maintaining their initial career choice although back-up plans were made or disregarding the possible influence of JIA on their career. Participants mentioned that in some cases initial choices of career or further education were abandoned due to JIA because of the prospect of performing heavy physical work, for example joining the military force or becoming a teacher of physical education. The majority of the participants reported that JIA did not influence their career choice. This was described in various ways. Feeling passionate about a career choice was mentioned as a reason to consider, start or persevere in the chosen career path regardless of the complaints (quotations 39–40). Another way was to ignore the possible impact of JIA in work participation (quotations 41–42).

### **Expectations**

Associated interview excerpts can be found in Table 5.

**Table 5:** Expectations, subtheme and associated interview excerpts

Quotation	Illustrative quotes	Participant nr
***Subtheme Work participation***		
43	There is a chance it’ll pass, so naturally, I hope that’ll be the case, but I don’t know. It may subside in the beginning and then come back a few months later, it’s highly unpredictable.	22
44	One step at a time and don’t plan ahead. It is quite hard to predict what I’ll be able to do.	21
45	I got the advice to search for an alternative study or route if I won’t be admitted, but I haven’t looked into it yet. First, I’ll focus on this year and then the next, then we’ll see. Not too far ahead.	22
46	I might not be able to stand for a long period or my knees and feet might hurt after a long day of work. But I’ll see what happens, I’m not worrying about it too much.	6
47	Actually, I think I won’t be able to continue working for forty hours a week. But at this point, I don’t want to think about it, we’ll see what happens.	3
48	Hopefully without pain. I hope I will have graduated and haveg a nice job. I hope I will be able to travel a lot as well.	21
49	I’ll have my own company. And I would like to organize charity events, also abroad, for people who have a disability.	11
50	I’ll have graduated from this programme and have started the next, I might have graduated from that one as well. And by that time, I want to have two children and be married.	9

### Work participation

Expectations of patients with JIA regarding future work participation were expressed as hopes and dreams rather than predictions. The uncertainty how JIA would develop was reported as a reason for having difficulty to plan their work/career and formulate expectations (quotation 43). Additionally, participants mentioned that because of the variable disease course, it was difficult to plan ahead, (quotation 44). When explicitly asked, participants acknowledged the possibility that JIA might complicate work participation, but did not reflect on any potential challenges in future work. Participants reported a “Just wait and see” principle (quotation 45–47).

When participants reflected on the more distant future, optimistic answers were given concerning their future work and home situation. Most participants did not mention JIA as being part of their future, nor as an influence in their future career. For examples, see quotations 48–50.

## Discussion

In this study we researched experiences of adolescents with JIA during school life, including leisure activities and at a part-time job, and the perspectives and expectations regarding their future work participation. Individual interviews were held with 22 adolescents diagnosed with JIA with different social, economic and educational backgrounds. The experiences of having JIA at school, sports or at a job show varying degrees of understanding and support as well as differences in adjustments that were provided. The perspectives of adolescents with JIA on future work participation described how participants saw themselves as students and employees with JIA and how they wished to be seen. Expectations could not always be formulated due to the variable course of JIA. Regarding the more distant future, career expectations were optimistic.

Within participants’ perspectives the theme ‘normality’ and the reasons for not disclosing having JIA were highly intertwined and can be seen as pursuing an equal position as compared to ‘healthy’ others.

This is mentioned in earlier studies. For example, in a systematic review of Tong et al [26] it was referred to as an “aversion to being different” and “striving for normality””, the latter including “preserving social identity”. Another qualitative study by Hanson et al. [27] mentioned that one of the reasons not to disclose having JIA is “not wanting to be perceived as different”. This is comparable to our results since participants who did not previously disclose having JIA or did not want to do so in the future, were trying to prevent becoming known as ‘the JIA patient’. This is probably the reason why adjustments in workload at a part-time job were rarely formally implemented or requested. Colleagues, often friends or family members, helped out when necessary, sometimes without the employer knowing. This corresponds to the study of Hanson et al. [27] which noted a restricted disclosure, e.g. confiding a trusted co-worker. Another reason mentioned in our study for not disclosing having JIA is negative experiences with educators or employers. This was said to result in a lack of confidence and trust in future employers.

Nondisclosure is a cause of concern since earlier research in the adult population stated that not disclosing having a chronic disease may result in unrealistic expectations by employers and workers not being able to meet their job demands [28]. Although the participants preferably didn’t want others to know they have JIA, they presumed that disclosure is necessary to receive consideration or the needed support from their fellow students, teachers and (future) colleagues and employers. Adolescents seem to waver between disclosure and nondisclosure. Eagerness to be normal and the high level of perseverance were revealed as possible pitfalls of nondisclosure.

Assuming perspectives and expectations are based on experiences, it is surprising that having JIA did not lead to concern about its potentially significant role in the participants’ career choice. Participants made their career choice without appreciating the experienced impact of JIA on participation in school or part-time employment in the past. A similar pattern is seen regarding the attitude the participants held towards adjustments. Although participants where in need for a special chair or an adjusted schedule at school or at a part time job, most participants did not take adjustments into account in their future work. Even when participants experienced several setbacks due to JIA, which were reflected upon during the interviews, motivation for a certain career path seemed to overrule choosing an alternative. This suggested that career decisions were rather based on aspirations (hopes and dreams) than on expectations (possibilities and challenges).

This result confirmed earlier research which stated that plans after high school of patients with JIA did not deviate from healthy peers [7]. Hanson et al. [27] also mentioned that some young people were highly committed to their career choice and willing to overcome barriers caused by JIA. On the other hand, it contradicts a study by Walter et al. [29] which showed around half of their participants (*n* = 154, mean age 20) had taken JIA into account when choosing their career. Additionally, Tong et al. [26] identified the subtheme ‘resourcefulness’, roughly meaning accepting limitations and seeking alternative activities in leisure activities and vocational opportunities.

With this study, we purposively chose this age category to give insight in the perspectives before entering the labour market. Consequently we assume not all participants had reached vocational adaptability. Still, not considering JIA in future career planning seems a rather naïve attitude which appears to be in close association with the hope or belief that JIA will subside. Unfortunately many patients will not achieve remission, although the remission rate increases with disease duration, from 7% at 18 months, 40% after a minimum of 10 years [4] up to 59% after a follow-up of 30 years [30]. Another reason mentioned for patients not to consider JIA in their future plans, is the variability in disease course, which, at this point, cannot be predicted [3]. RA is comparable in its variability. A study on behavioural coping in RA showed that letting the disease influence the choice of a job was a high preventive factor in withdrawal from the labour force [31]. This underlines the need for discussing vocation with adolescents with JIA.

Transition includes disease education and support in self-management [32, 33]. The influence of the disease on everyday life and how this changes while becoming an adult, is thought to be equally important [34–37]. The European League Against Rheumatism / Paediatric Rheumatology European Society (EULAR/PReS) considers the subject of vocation part of holistic care and therefore an important subject in the transition process towards adult care. Discussing vocational aspects with patients such as developing skills in disclosure and career prospects, are therefore recommended [38]. Based on our study we advise healthcare professionals to start this conversation in early adolescence. Thus creating awareness for the barriers and opportunities in future work participation before choices are made regarding career planning.

## Conclusion

This study showed a range of experiences, perspectives and expectations of adolescents with JIA regarding future work participation. Differences were seen in the support that adolescents received in dealing with JIA at school, leisure activities and work. Varying approaches were mentioned on how to pursue a desired vocation. Surprisingly, participants often disregarded having JIA when making plans for their future career. Appreciating the difference in vocational aspirations and expectations will contribute to the vocational adaptability of adolescents with JIA. Facilitating an open discussion about the possible limitations accompanying JIA with (future) educators and employers might prevent overburden and increase the chance of starting a career which would accommodate the patient with JIA in the near and distant future.

## Data Availability

The datasets comprise full text interviews in Dutch and patient characteristics. The datasets analysed during the current study are available from the corresponding author on reasonable request.

## Abbreviations

CHAQ: Child Health Assessment Questionnaire
COREQ: Consolidated criteria for reporting qualitative research
ERA: Enthesitis related arthritis
EULAR: European League Against Rheumatism
ILAR: International League Against Rheumatism
JIA: Juvenile idiopathic arthritis
PedsQL: Pediatrics Quality of Life inventory
PReS: Paediatric Rheumatology European Society
RF: Rheumatoid factor
